# Recurrent Ileo-Ileal Intussusception in Early Infancy Requiring Surgical Rescue: A Report of a Rare Case

**DOI:** 10.7759/cureus.106546

**Published:** 2026-04-06

**Authors:** Sania Shahid, Sulaima Taji, Saif M Khan, Mina Almanasir, Nusrath M P, Sana Ahmed, Mariam Al Ali, Aqsha Shahid

**Affiliations:** 1 Pediatric Emergency Medicine, Al Jalila Children's Specialty Hospital, Dubai, ARE; 2 Medicine, Tbilisi State Medical University, Tbilisi, GEO; 3 Medicine, Dubai Medical University, College of Medicine, Dubai, ARE; 4 Emergency Medicine, Dubai Health, Mohammed Bin Rashid University (MBRU), Dubai, ARE; 5 Medicine, University of Georgia, Tbilisi, GEO

**Keywords:** bilious vomiting, ileo-ileal intussusception, infantile colic, intussusception, paediatric intestinal obstruction, pediatric emergency department (ped), unexplained abdominal pain

## Abstract

Intussusception is an important cause of intestinal obstruction in infants, most commonly involving the ileocolic region. Nonoperative reduction using air or contrast enemas is the first-line management; however, recurrence or failed reduction may require surgical intervention. We report a case of a two-month-old female who developed three episodes of recurrent ileo-ileal intussusception over a three-week period. Her early presentations were initially attributed to a nonspecific gastrointestinal illness, posing a diagnostic challenge, and definitive surgical intervention was eventually required because of ongoing symptoms. This case highlights the diagnostic challenges of recurrent intussusception in early infancy and emphasizes the need for timely surgical consideration following failed nonoperative management.

## Introduction

Intussusception, the invagination of bowel into an adjacent segment, is the leading cause of intestinal obstruction in infants [1,2]. Although typically seen in children aged six to 36 months, presentation in younger infants is uncommon and often atypical, increasing the risk of delayed diagnosis and recurrent episodes [3,4]. Classical symptoms include intermittent abdominal pain, vomiting, and "currant jelly" stools, although neonates and young infants may exhibit nonspecific signs such as irritability or isolated vomiting [5]. Ultrasound remains the diagnostic modality of choice, while therapeutic air or contrast enemas provide both diagnostic confirmation and potential resolution [6]. Radiographic techniques, including fluoroscopy and barium enema, retain diagnostic and therapeutic value in select settings where ultrasound access or operator expertise is limited [7]. Although air enema reduction is successful in up to 80% to 90% of cases, recurrence occurs in approximately 10% of patients, often within 24 to 48 hours, but may also be delayed [8]. Recurrent or persistent intussusception may require surgical reduction, particularly in cases of failed enema attempts or suspected pathologic lead points [9].

## Case presentation

A female infant, born full term via uncomplicated vaginal delivery, presented twice to the emergency department with episodes of regurgitation and non-bilious vomiting. As the infant was thriving on exclusive breastfeeding with no concerning clinical findings, the symptoms were initially attributed to physiological gastroesophageal reflux. At six weeks of age, she presented for the third time with persistent vomiting that had become bilious and was associated with irritability.

**Figure 1:**
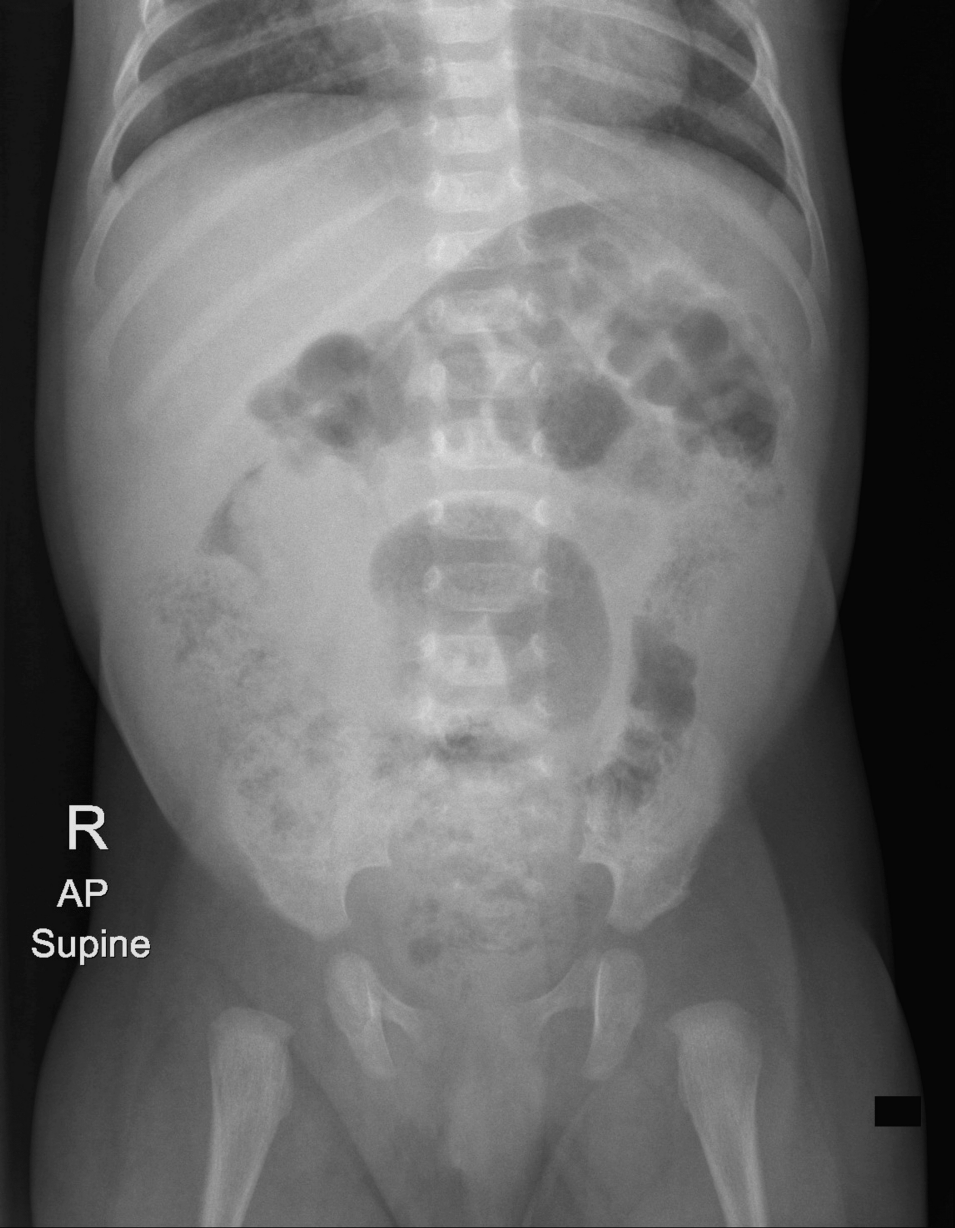
Abdominal X-ray shows a dilated central tubular bowel loop. The colon is loaded with fecal matter.

**Figure 2:**
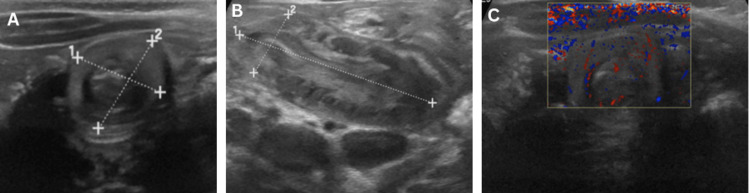
First episode of ileo-ileal intussusception. (A) Transverse ultrasound view demonstrating the classic target sign. (B) Longitudinal view showing pseudokidney appearance consistent with ileocolic intussusception. (C) Doppler image demonstrating preserved bowel vascularity.

**Figure 3:**
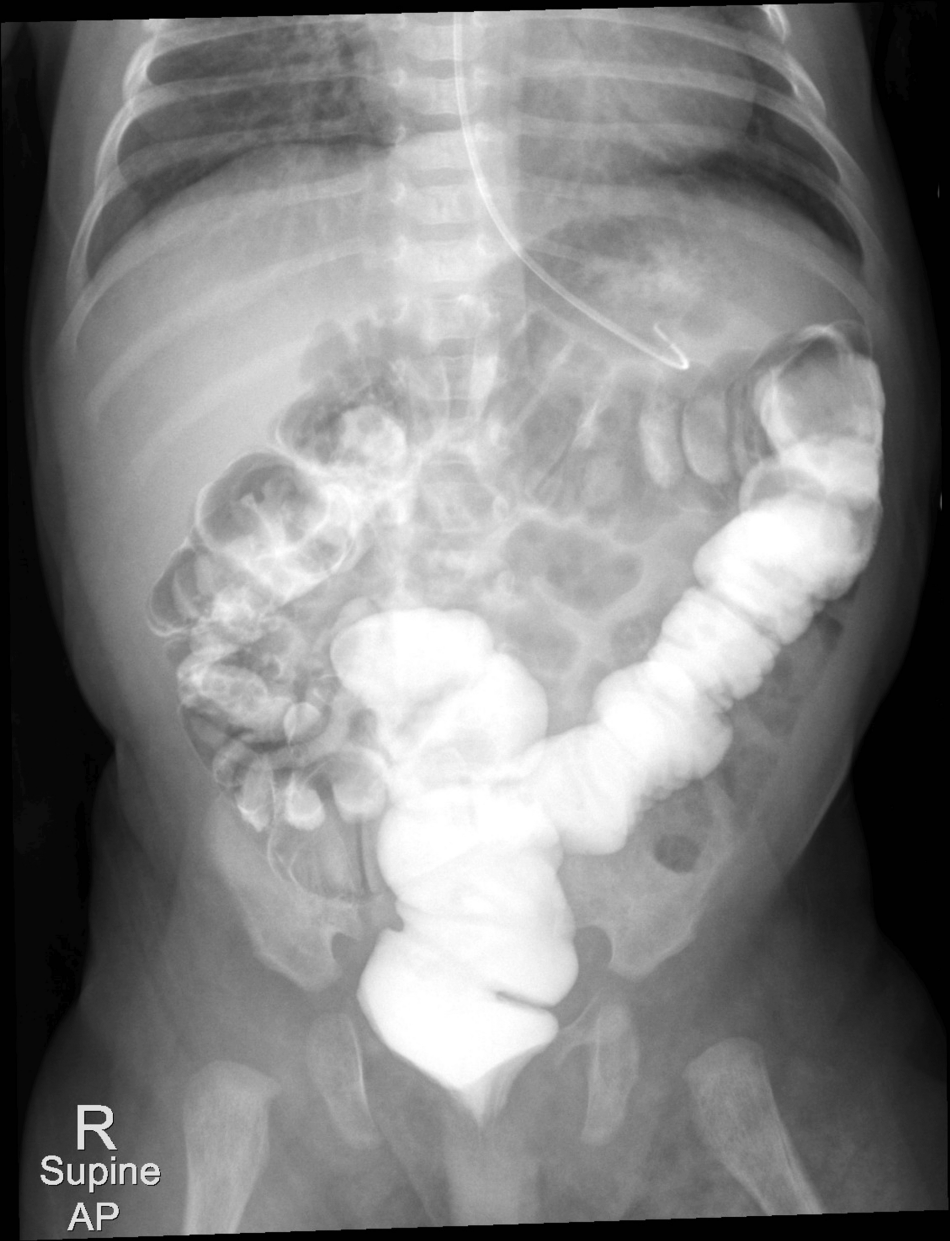
Upper gastrointestinal study of barium meal with small bowel follow-through shows normal location and caliber of the small bowel loops. A delayed image shows the passage of contrast into the large bowel with the normal position of the cecum. No malrotation of the midgut and no evidence of bowel obstruction was noted.

An abdominal radiograph was suspicious for intestinal obstruction, but was inconclusive (Figure 1). This prompted further evaluation with abdominal ultrasonography, which demonstrated a target sign with a pseudokidney appearance, findings suggestive of ileo-ileal intussusception (Figure 2). An urgent surgical consultation was obtained. In view of the patient’s age and bilious vomiting, the surgical team recommended further contrast imaging to exclude other potential surgical causes of obstruction in early infancy, including malrotation with midgut volvulus and other structural gastrointestinal anomalies. The contrast study was normal, with no evidence of malrotation or obstruction (Figure 3).

The infant remained clinically stable and tolerated feeds during observation, following which she was discharged with close outpatient follow-up.

**Figure 4:**
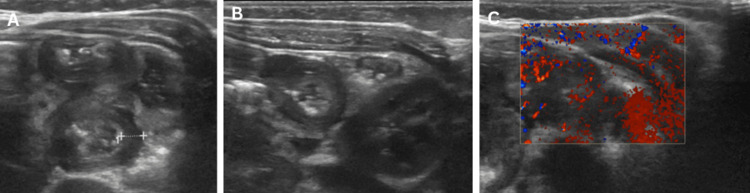
Second episode of ileo-ileal intussusception. (A) and (B) Transverse ultrasound views showing target sign indicating recurrence. (C) Doppler image demonstrating preserved bowel vascularity.

One week later, at seven weeks of age, she was again seen for non-bilious vomiting and irritability. Abdominal ultrasound revealed a classic “target sign” in the right lower quadrant (Figure 4), consistent with long-segment ileo-ileal intussusception. This was managed with observation alone, with a repeat scan after three hours demonstrating resolution. The child was monitored for 24 hours and discharged in stable condition.

**Figure 5:**
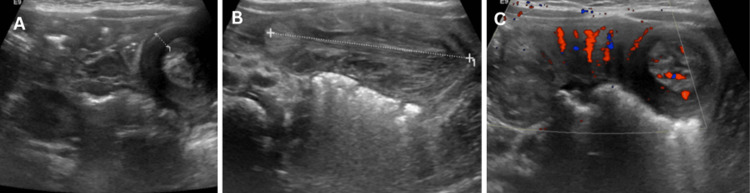
Third episode of ileo-ileal intussusception. Findings are seen unchanged when compared to the previous scan. (A) Target sign with bowel wall edema with a long segment of small bowel intussusception seen extending from the left upper quadrant to the right lower abdominal quadrant, about 2.5 cm in short axis and 6 cm in length. (B) Pseudokidney appearance with mild wall thickening of the involved bowel loops is noted. (C) Blood flow was seen maintained on color Doppler along the layer of the bowel loops and within the surrounding mesentery.

**Figure 6:**
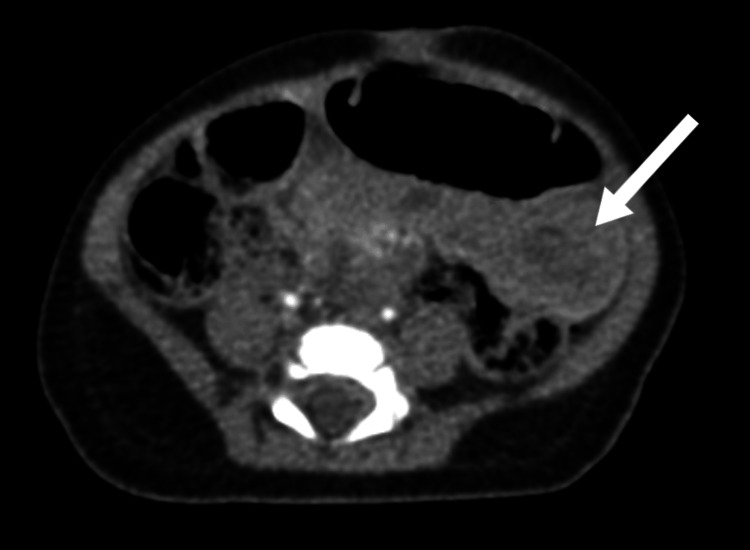
Axial CT of the mid-abdomen demonstrates ileo-ileal intussusception (white arrow). The walls of the bowel loops involved in the intussusception are thickened, showing average mural enhancement. No obvious lead point could be identified. It measures about 7.5 cm in length and 2.7 cm in short axis.

However, within a week of discharge, at eight weeks of age, the infant re-presented with recurrent daily vomiting for a few days that had again turned bilious. As expected, a recurrence of the long segment ileo-ileal intussusception was picked up on ultrasound of the abdomen (Figure 5), and a CT scan with contrast was ordered to decide whether a lead point is involved; however, no obvious points were delineated, as shown in Figure 6. In view of recurrent intussusception with persistent symptoms and the absence of an identifiable lead point on prior episodes, surgical exploration was performed to identify the underlying etiology. Intraoperatively, a polyp was identified in the mid-ileum to have been the lead point, so the patient underwent resection and anastomosis of the involved segment, which was 1.3 cm in length.

Postoperatively, the infant recovered uneventfully, resumed full feeds, and had normal bowel movements. Histopathology of the polyp showed inflammatory changes but was negative for malignancy. The patient was discharged home on postoperative day six and remained asymptomatic on follow-up.

## Discussion

Intussusception in infants younger than three months is rare and often presents atypically, which can contribute to delayed diagnosis [4,5]. In our patient, the first episode of excessive crying and vomiting was presumed to be nonspecific gastroenteritis, and abdominal radiography failed to reveal obstructive signs. The absence of classic findings such as palpable abdominal mass or currant jelly stools early in the course can further obscure diagnosis [10].

Ultrasound remains the most sensitive modality, with sensitivity approaching 98% for intussusception [6,11]. Once diagnosed, a pneumatic or hydrostatic enema is the initial treatment of choice [2]. However, reduction failure rates may be higher in younger infants and in those with prolonged symptoms or recurrent episodes. Factors contributing to failed enema reduction include prolonged symptom duration, significant mucosal edema, and younger age, which may reduce responsiveness to pneumatic pressure [8,12].

Although most recurrences occur within 24 to 48 hours, delayed recurrence beyond one week, such as in this case, underscores the need for close outpatient follow-up even after initial resolution [13]. Surgical intervention is warranted when nonoperative management fails or when a pathological lead point is suspected. While lead points are more common in older children, up to 10% of recurrent cases may harbor such findings [14,15]. In our case, a lead point was identified. Appendectomy was also performed as a precaution to avoid future diagnostic confusion. Prompt surgical management led to a successful outcome.

A pathological lead point should be considered in cases of recurrent intussusception [5]. Polyps, though uncommon in infants, are a recognized cause and are more frequently seen in older children [5,16]. The most common types include juvenile polyps, which typically occur between two and five years of age, and they most often arise in the colon [16]. When present, polyps can act as a lead point for intussusception, particularly in ileocolic or colocolic types [16]. In younger infants, identification of a lead point is less common; however, recurrent or atypical presentations should prompt further evaluation for an underlying pathology, as demonstrated in this case.

## Conclusions

This case underscores the diagnostic complexity of intussusception in infants younger than three months, in whom symptoms are frequently nonspecific and classical clinical features may be absent. Recurrent ileo-ileal intussusception beyond the typical early recurrence window further highlights the need for sustained clinical vigilance even after successful enema reduction. In young infants presenting with persistent or recurrent vomiting, irritability, or hematochezia, early ultrasonographic evaluation should be strongly considered despite initially reassuring findings. Moreover, failure of repeat nonoperative reduction should prompt timely surgical intervention to prevent bowel compromise and associated morbidity. A heightened index of suspicion, close follow-up after initial reduction, and a low threshold for surgical consultation are critical to optimizing outcomes in this vulnerable population.

## References

[1] Guo H, Lei H, Luo J, Yang J, Bian H, Yang H, Guo Q (2025). Clinical manifestation and treatment of intussusception in children aged 3 months and under : a single centre analysis of 38 cases. BMC Pediatr.

[2] Madan AJ, Haider F, Alhindi S (2021). Profile and outcome of pediatric intussusception: a 5-year experience in a tertiary care center. Ann Pediatr Surg.

[3] Shavit I, Levy N, Dreznik Y, Soudack M, Cohen DM, Kuint RC (2024). Practice variation in the management of pediatric intussusception: a narrative review. Eur J Pediatr.

[4] Gray MP, Li SH, Hoffmann RG, Gorelick MH (2014). Recurrence rates after intussusception enema reduction: a meta-analysis. Pediatrics.

[5] Adhikari S, Koirala DP, Pokhrel RP, Dahal GR, Kharel S, Neupane S (2022). Risk factors for recurrent intussusception after successful reduction in pediatric patients in a tertiary care hospital of Nepal: a prospective study. Ann Med Surg (Lond).

[6] Ye X, Tang R, Chen S, Lin Z, Zhu J (2019). Risk factors for recurrent intussusception in children: a systematic review and meta-analysis. Front Pediatr.

[7] Otero HJ, De Leon Benedetti LS, Applegate KE (2024). Intussusception in children: diagnostic imaging and treatment. Evidence-Based Imaging in Pediatrics. Evidence-Based Imaging.

[8] Klimeczek Chrapusta M, Preinl M, Łubniewska Z, Procháska F, Gruba M, Górecki W (2024). Predictive factors for failure of nonsurgical management of intussusception and its in-hospital recurrence in pediatric patients: a large retrospective single-center study. Egypt Pediatric Association Gaz.

[9] Guo JY, Qian YF (2022). Predicting recurrent cases of intussusception in children after air enema reduction with machine learning models. Pediatr Surg Int.

[10] Kotb M, Abdelatty M, Rashwan H, AbdelMeguid Y, Elrouby A (2021). Intussusception in preterm neonates: a systematic review of a rare condition. BMC Pediatr.

[11] Soleimanpour Z, Memarian S, Rajabi MM, Zamani Z, Alimadadi H, Gharib B (2025). Clinical and paraclinical differences between pediatric patients requiring surgical versus non-surgical treatment for intussusception: a retrospective study at a referral center in Iran. BMC Surg.

[12] Byrne AT, Geoghegan T, Govender P, Lyburn ID, Colhoun E, Torreggiani WC (2005). The imaging of intussusception. Clin Radiol.

[13] Williams JL, Woodward C, Royall IR (2022). Outcomes in pediatric patients with documented delays between ileocolic intussusception diagnosis and therapeutic enema attempt: evaluation of reduction efficacy and complication rate. Emerg Radiol.

[14] Navarro O, Dugougeat F, Kornecki A, Shuckett B, Alton DJ, Daneman A (2000). The impact of imaging in the management of intussusception owing to pathologic lead points in children. A review of 43 cases. Pediatr Radiol.

[15] Fernandes EG, Leshem E, Patel M, Flannery B, Pellini AC, Veras MA, Sato HK (2016). Hospital-based surveillance of intussusception among infants. J Pediatr (Rio J).

[16] Kay M, Eng K, Wyllie R (2015). Colonic polyps and polyposis syndromes in pediatric patients. Curr Opin Pediatr.

